# Using *Drosophila* as an integrated model to study mild repetitive traumatic brain injury

**DOI:** 10.1038/srep25252

**Published:** 2016-05-04

**Authors:** Ayeh Barekat, Arysa Gonzalez, Ruth E. Mauntz, Roxanne W. Kotzebue, Brandon Molina, Nadja El-Mecharrafie, Catherine J. Conner, Shannon Garza, Girish C. Melkani, William J. Joiner, Marta M. Lipinski, Kim D. Finley, Eric P. Ratliff

**Affiliations:** 1 Donald P. Shiley BioScience Center, San Diego State University, San Diego, CA, USA; 2 Department of Biology, San Diego State University, San Diego, CA, USA; 3 Department of Pharmacology, University of California San Diego, La Jolla, CA, USA; 4 Shock, Trauma, and Anesthesiology Research (STAR) Center; Department of Anesthesiology, University of Maryland School of Medicine, Baltimore, MD, USA; 5 Department of Chemistry, San Diego State University, San Diego, CA, USA

## Abstract

Traumatic brain injury (TBI) is a major cause of morbidity and mortality worldwide. In addition, there has been a growing appreciation that even repetitive, milder forms of TBI (mTBI) can have long-term deleterious consequences to neural tissues. Hampering our understanding of genetic and environmental factors that influence the cellular and molecular responses to injury has been the limited availability of effective genetic model systems that could be used to identify the key genes and pathways that modulate both the acute and long-term responses to TBI. Here we report the development of a severe and mild-repetitive TBI model using *Drosophila*. Using this system, key features that are typically found in mammalian TBI models were also identified in flies, including the activation of inflammatory and autophagy responses, increased Tau phosphorylation and neuronal defects that impair sleep-related behaviors. This novel injury paradigm demonstrates the utility of *Drosophila* as an effective tool to validate genetic and environmental factors that influence the whole animal response to trauma and to identify prospective therapies needed for the treatment of TBI.

Traumatic brain injury (**TBI**) is a major cause of morbidity and mortality worldwide that disproportionately impacts children and young adults. Most studies modeling TBI focus on the damage and repair mechanisms that follow a single, high-impact traumatic event^1^. Recently, there has been growing interest in milder forms of TBI that involve low-impact multi-bout injuries like those associated with contact sports or domestic violence^2^,^3^,^4^,^5^. In those cases, obvious symptoms like coma, amnesia or loss of consciousness are often relatively mild or absent^4^. However, recent studies examining professional athletes have highlighted the occurrence of subtle long-term morphological changes in the brain that can follow a series of low-impact traumatic injury events^5^,^6^,^7^. This includes the progressive development of chronic traumatic encephalopathy (CTE), which is often associated with the deposition of hyperphosphorylated Tau protein in neurological tissues^5^. In addition, mild TBI can also be associated with long-term changes to behavior and mood, which includes cognitive deficits, depression and altered sleep patterns^4^,^6^,^7^. The implications are that even mild injuries to the brain can generate progressive defects that are difficult to detect until considerable time has elapsed.

The acute and long-term neurological damage caused by TBI are intense areas of study. What has become clear is that the outcome of brain trauma not only reflects the direct cellular damage caused by the primary impact, but also involves the cascade of secondary cellular and molecular responses that are activated following the initial injury^8^. These responses include the rapid initiation of inflammatory pathways, which are necessary to initiate host defense responses and to remove cellular debris^9^. However, the inability to appropriately attenuate these responses can lead to pathological inflammation that can further damage cells and tissues, thus worsening long-term outcomes^10^. In addition, intracellular clearance pathways that remove damaged components are essential to facilitate cellular repairs following injury^11^. One such mechanism is macroautophagy (hereto after referred to as autophagy). This process promotes the sequestration and elimination of extraneous or damaged intracellular materials via a highly conserved lysosomal-dependent clearance pathway^12^,^13^. Markers of autophagy are increased following TBI and recent work has shown that this is due to defects in lysosomal function, which leads to the accumulation of dysfunctional autophagosomes^11^,^14^. This impairment likely contributes to negative outcomes following TBI exposure.

The individual TBI outcomes are often highly heterogeneous, due to variations in the location and extent of the primary damage. However, this heterogeneity not only reflects the complexity of the nervous system, but also includes genetic and environmental factors that influence the cellular and molecular responses to damage^8^,^15^. Another important factor is age, with older individuals typically having more serious complications and worse outcomes following TBI^16^. Unfortunately, systematically assessing the impact of these factors is difficult in the commonly used rodent models of TBI, due to the prohibitively high costs of large-scale screens in rodents and their relatively long life spans. Therefore, there is a need to develop a TBI model in a simpler organism that would allow for rapid and cost effective identification of genetic, environmental and age-dependent factors influencing injury outcomes. Indeed, recent studies have shown that novel TBI models using lower organisms can recapitulate some of the phenotypes observed in mammalian systems^17^,^18^.

Here we describe the development of both a high-impact and mild repetitive traumatic brain injury model using adult *Drosophila melanogaster*. With this system, we have reproducibly delivered traumatic injury to adult flies and demonstrated dose-dependent alterations to longevity profiles and the sleep/wake cycle. At the cellular level, injured flies showed a dynamic activation of inflammatory markers and the accumulation of autophagosomes in neural tissues following injury. Overall, our results in *Drosophila* parallel findings from humans and other mammalian TBI studies. Our unique injury paradigm will facilitate the systematic characterization of genetic, age-related and environmental factors that can influence the acute and long-term response to neural injury.

## Results

### Establishing a *Drosophila* model of TBI

**Figure 1:**
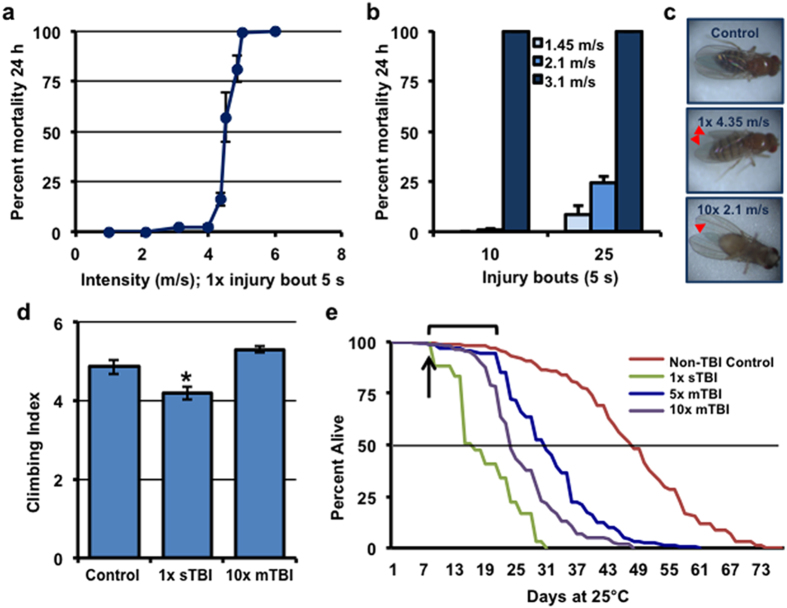
Development of the mild TBI model in *Drosophila*. (**a**) Male wild-type flies (1-week of age) were subjected to different injury intensities (5-second) using the Omni Bead Ruptor-24 and the number of dead flies (n ≥ 3; cohorts of 50 flies) was used to establish the 24 hour post-injury mortality. (**b**) Male flies (1-week of age) were subjected to multiple 5-second injury bouts at lower intensity settings and used to establish 24 hour post-injury mortality for multi-bout injuries (n ≥ 3; cohorts of 50 flies). (**c**) Representative images of flies following injury highlighting external damage to wing structures (red arrows). (**d**) The climbing index of groups of male wild-type control flies and flies subjected to a single sTBI (**1×**, 4.35 m/s intensity) or **10×** mTBI (2.1 m/s intensity) injury bouts (1-week of age; cohorts of 25 flies; n ≥ 6). (**e**) Lifespan profiles of male wild-type control flies and flies subjected to **1×** (4.35 m/s intensity), **5×** or **10×** (2.1 m/s intensity) injury bout(s) at 1-week of age (n ≥ 59, arrow indicates time of injury). The bracket highlights the 2-week delay in mortality in flies exposed to mTBI. See Materials and Methods for clarification of the injury protocol and Supplementary Table S1 for additional lifespan data and statistics. *P < 0.05.

To establish a model of traumatic injury using adult *Drosophila*, we developed a novel delivery method to generate highly reproducible levels of trauma to large numbers of flies. Using the Omni Bead Ruptor-24 Homogenizer platform, we subjected flies to a defined injury paradigm by modulating the intensity of injury (meters/second, [m/s]), duration of injury (s), and the number of injury bouts. Briefly, 10 flies were placed into empty 2-ml screwcap tubes that were secured individually into the Omni Bead Ruptor-24 system. To optimize traumatic injury conditions, separate cohorts of flies were subjected to a single 5-second injury bout with varying intensities. We found intensities of 5.0 m/s and higher were acutely lethal, resulting in nearly 100% mortality within 24 hours (Fig. 1a). In contrast, exposure to a 4.35 m/s intensity bout resulted in a ~10–25% survival, while at lower intensities nearly all flies survived after 24 hours (Fig. 1a). Given the acute mortality of flies at 4.35 m/s intensity, the high impact or severe TBI (**sTBI**) studies were standardized to this intensity. These data were also used to develop a model to study the long-term effects of low impact or mild traumatic brain injury (**mTBI**) on neural function in *Drosophila*. Repetitive, mild TBI is relatively common and can be experienced by individuals participating in activities such as contact sports (*i.e.* mild repetitive TBI)^19^. To assess the acute mTBI response, we measured the 24-hour mortality of flies subjected to multiple injury bouts at sub-lethal intensities (1.45–3.1 m/s). Flies subjected to 25 injury bouts (30-second rest between bouts) exhibited high levels of mortality regardless of the intensity. However, flies exposed to 10 injury bouts at lower intensity levels (1.45–2.1 m/s) showed little or no lethality within 24 hours (Fig. 1b). In fact, following this injury regimen, the majority of the flies exhibited normal locomotor behaviors within 10 minutes. Therefore, we standardized our multi-bout mTBI studies to the 2.1 m/s intensity.

Flies exposed to a single sTBI (4.35 m/s) bout and flies exposed to 10 mTBI (2.1 m/s) bouts both exhibited minimal external defects, with the exception of minor breaks or tears to delicate structures of the wing (Fig. 1c). Of note, the legs and other external structures of flies from both injury cohorts remained largely intact (Fig. 1c). Since the mTBI treated flies appeared externally normal, we initially assessed the extent of internal damage by examining the negative geotaxis response of the flies after exposure to the injury regimens. The negative geotaxis response is a highly reproducible climbing behavior in adult *Drosophila* that involves the coordinated responses between the neural and skeletal muscle tissues^20^,^21^. The climbing abilities of non-injured controls, sTBI (1×, 4.35 m/s) and mTBI (10×, 2.1 m/s) treated fly cohorts were assessed 2-day following exposure to trauma (Fig. 1d). Both control and mTBI (10×, 2.1 m/s) treated flies exhibited similar climbing indexes^21^. Flies exposed to sTBI (1×, 4.35 m/s) exhibited a modest, but significant, reduction in climbing abilities, suggesting that they sustained a modest level of damage to internal structures and tissues (Fig. 1d). As a preliminary assessment, these data suggest that mTBI-treated flies exhibited phenotypes consistent with those presented by people who have experienced mild levels of injury or trauma (*i.e.* minimal visual or early cognitive signs of damage)^22^.

To examine the long-term impact of the different traumatic injury regimens, we examined longevity profiles. Following a single sTBI bout (4.35 m/s) at 1-week of age, flies exhibited increased mortality within 24 hours and an overall 60% reduction in mean longevity when compared to non-injured cohorts (Fig. 1e, Supplementary Table S1). Flies exposed to 5 or 10 bouts of mTBI (2.1 m/s) had a more modest reduction in average lifespan profiles, which demonstrated that the number of mTBI bouts had a negative dose-dependent effect on longevity (35% and 45% reduction respectively, Fig. 1e, Supplementary Table S1). An additional feature highlighted by mTBI longevity profiles was the nearly two week delay in mortality following injury (bracket, Fig. 1e). This delay was not demonstrated by flies exposed to a single sTBI (4.35 m/s) bout or by middle-aged flies (3-weeks old) treated with standard mTBI (10×, 2.1 m/s) conditions (Supplementary Fig. S1, Table S1). The increase in acute mortality of middle-aged flies (Supplementary Fig. S1, Table S1) is consistent with human and mouse studies that demonstrate worse outcomes for older individuals following TBI^16^,^23^.

### Neural-specific changes following mTBI

**Figure 2:**
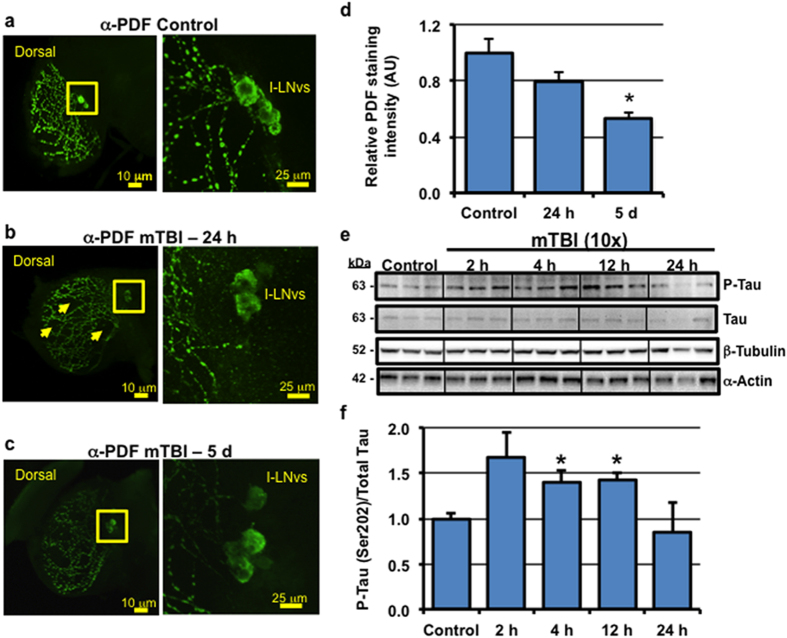
Acute impact of mild TBI on neurons. (**a–c**) The brains of adult male wild-type flies were dissected from (**a**) control flies (n = 15) and mTBI-treated (10×, 2.1 m/s) flies (**b**) after 24 hours (n = 11) and (**c**) 5 days (n = 9). Representative compressed confocal Z series and enlarged images (inset) of PDF staining patterns show the l-LNvs cell bodies and innervation patterns into the adult optic lobes. Arrows highlight damaged CNS regions that have lost PDF positive projections and synapses. (**d**) Quantification of PDF-staining intensity within the optic lobe (n ≥ 4 compressed Z-stacks of individual whole fly brains). (**e**) Replicate cohorts of male F1 flies with neuronal expression of the human Tau protein (APPL-Gal4/UAS-hTau) were exposed to **10×** mTBI (2.1 m/s intensity) bouts at 1-week of age and collected at indicated time points. Western blots of phosphorylated-Tau (Ser 202), total Tau, α-Actin, and β-Tubulin from neural lysates of control and mTBI-treated flies. (**f**) Quantification of the results in (**e**), normalized to total Tau protein levels. *P < 0.05.

Using our Bead Ruptor platform, mTBI-treated flies sustained multiple impacts across various parts of the body, including the head (Supplementary Video). To assess if mTBI results in structural damage to *Drosophila* neurons, we examined the morphology of cells that secret pigment dispersing factor (**PDF**). These cells represent a well-defined subset of neurons that are involved with the production and cyclical release of the fly circadian neuropeptide hormone PDF^24^. The limited number of PDF cells and their well-defined innervation patterns makes these neurons ideal subjects for image-based studies to detect morphological changes that result from trauma^25^. Under non-injured conditions, PDF-staining highlights the neuronal cell bodies (soma) in the central brain and the axons and presynaptic terminals in the optic lobes of adult *Drosophila* (Fig. 2a). Twenty-four hours following mTBI (10×, 2.1 m/s) exposure, the soma of large ventral lateral clock neurons (l-LNv) exhibited structural defects that included the absence of entire axonal and synaptic tracts within the optic lobe (arrows, Fig. 2b). Structural defects were still present 5-days following trauma (Fig. 2c). To quantitatively assess whether mTBI (10×, 2.1 m/s) exposure induced neuronal damage, we measured the PDF staining intensity per optic lobe (Supplementary Fig. S2). mTBI exposure (10×, 2.1 m/s) resulted in the progressive loss in PDF-positive fluorescence (Supplementary Fig. S2), which led to a 50% decrease in intensity 5-days following trauma (Fig. 2d). While the gross morphological defects to PDF positive cells and structures were variable, these imaging-based studies underscored the initial assessment that neural specific damage had occurred to adult *Drosophila* neurons following mTBI exposure.

In addition to causing structural damage, we assessed endogenous synaptic protein profiles to determine whether exposure to the mTBI regimen altered other neuronal biochemical markers. We observed an acute increase in the pre-synaptic proteins bruchpilot (BRP), synapsin, and syntaxin and the post-synaptic protein discs-large (DLG), which resolved to baseline levels within 24 hours following injury (Supplementary Fig. S3). Furthermore, we also assessed whether mTBI-treated flies exhibited an increase in the phosphorylation status of the human microtubule-associated protein Tau^26^. The presence of hyper-phosphorylated Tau is a common pathological feature of individuals that have been exposed to multiple mild TBI events^5^. In addition to protracted changes, mammalian models have also highlighted an acute increase in phosopho-Tau protein levels in neural tissues following TBI^27^,^28^. To determine if the *Drosophila* mTBI model could recapitulate these findings, we used the Gal4/UAS system to express the human Tau (hTau) protein in adult fly neurons (APPL-Gal4 driver)^29^; the adult F1 offspring (APPL-Gal4/UAS-hTau) were exposed to 10 bouts of mTBI (2.1 m/s). Consistent with mammalian TBI models, there was a rapid increase in hTau phosphorylation (Ser 202) following injury, which was attenuated within 24 hours following trauma (Fig. 2e,f)^27^. Combined, the mTBI-induced alterations in Tau phosophorylation and synaptic proteins levels are consistent with the structural damage to neurons observed in these flies.

### Non-Neuronal Alterations following Trauma

**Figure 3:**
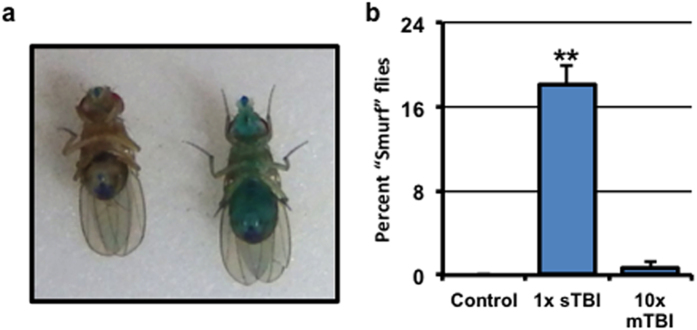
Intestinal integrity following TBI. (**a**) Representative image of a female “Smurf” fly that displayed intestinal barrier dysfunction, as detected by blue dye permeation throughout the body (*right*). When the intestinal barrier was intact, the blue dye is mainly found in the proboscis and the digestive tract (*left*). (**b**) The percent of male wild-type flies with intestinal barrier dysfunction in groups of control flies and flies subjected to a single sTBI (**1×**, 4.35 m/s intensity) or **10×** mTBI (2.1 m/s intensity) injury bouts (1-week of age; cohorts of 40 flies; n ≥ 6). **P < 0.01.

In addition to assessing neuronal defects, we assessed the integrity of the intestinal barrier in mTBI-treated flies. Disruption of the intestinal barrier has been shown to be a reliable predictor of age-associated and trauma-induced mortality in *Drosophila*^18^,^30^,^31^. Previous studies have demonstrated a non-toxic method to assess intestinal barrier dysfunction in adult flies by adding a non-absorbable blue dye to food and assessing whether the dye becomes dispersed throughout the fly body^30^,^31^. Systemically blue flies are referred to as “Smurfs” (Fig. 3a)^30^,^31^.

In this study, non-injured control flies and sTBI (1×, 4.35 m/s) and mTBI (10×, 2.1 m/s) treated fly cohorts were placed onto standard fly food containing blue dye. Less than 1% of the non-injured controls and the mTBI-treated flies exhibited systemic blue coloring within 24 hours following trauma (Fig. 3b). In contrast, 18% of the sTBI treated flies showed the Smurf phenotype within 24 hours following trauma exposure, which is reflected in the percentage of dead flies in this time period. Combined with normal negative geotaxis response profiles, these data suggest that mTBI-treated flies sustain modest levels of damage to non-neuronal tissues and structures.

**Figure 4:**
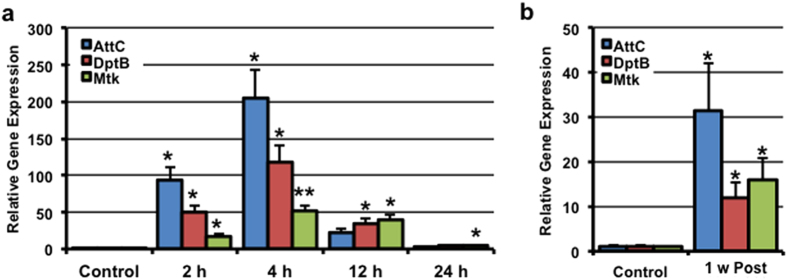
Innate immune response activation following mild TBI. Replicate cohorts of wild-type male flies (1-week of age; cohorts of 25 flies; n ≥ 3) were exposed to **10×** mTBI (2.1 m/s intensity) bouts. RNA was isolated from the heads of control and injured flies at the indicated time-points following mTBI during the (**a**) acute phase and at (**b**) 1 week post-injury (long-term). Expression of *AttC*, *DptB*, and *Mtk* were normalized using *Cyp1* as the reference gene. *P < 0.05, **P < 0.01.

There are multiple cellular/molecular pathways activated following human trauma and other mammalian TBI models. The rapid activation of innate immune system is a highly conserved, well-characterized response of tissues damaged following traumatic injury^32^. Inflammation plays a critical role in signaling damage and activating repair processes, often by recruiting immune cells to local regions of damage^8^,^9^. Once at an injury site, activated microglia, macrophages, and other immune cells facilitate the removal of dead or damaged cells and tissue debris^9^. In flies, the molecular mediators of the innate immune system are analogous to mammalian systems and consist of the Toll (Toll-like receptor) and the Imd pathways (Tumor necrosis factor receptor)^33^. Activation of these pathways promotes the up-regulation of subsets of anti-microbial peptides (AMP)^33^. Following mTBI treatment (10×, 2.1 m/s), flies showed an immediate and robust increase in the expression of the AMP genes *AttC*, *DptB*, and *Mtk* within the head, which peaked at 4 hours following injury and returned to near baseline levels by 24 hours (Fig. 4a). These findings were consistent with a previous *Drosophila* TBI model that demonstrated a rapid activation of several AMP genes in the head within 24 hours of injury^17^.

In addition to the acute induction following TBI, humans and other mammalian models often show a longer-term, secondary activation of the innate immune response following traumatic injury^34^. Therefore, we also assessed the AMP expression profile of mTBI treated flies (10×, 2.1 m/s) 1-week following trauma. Unexpectedly, injured flies exhibited a 12- to 30-fold increase in the expression of these AMP genes in the head 1-week following TBI exposure (Fig. 4b). Combined, these results indicate that *Drosophila* exposed to mild levels of trauma also exhibit alterations that are consistent with both the acute and long-term activation of inflammatory pathways following head injury.

### Autophagy and mTBI

**Figure 5:**
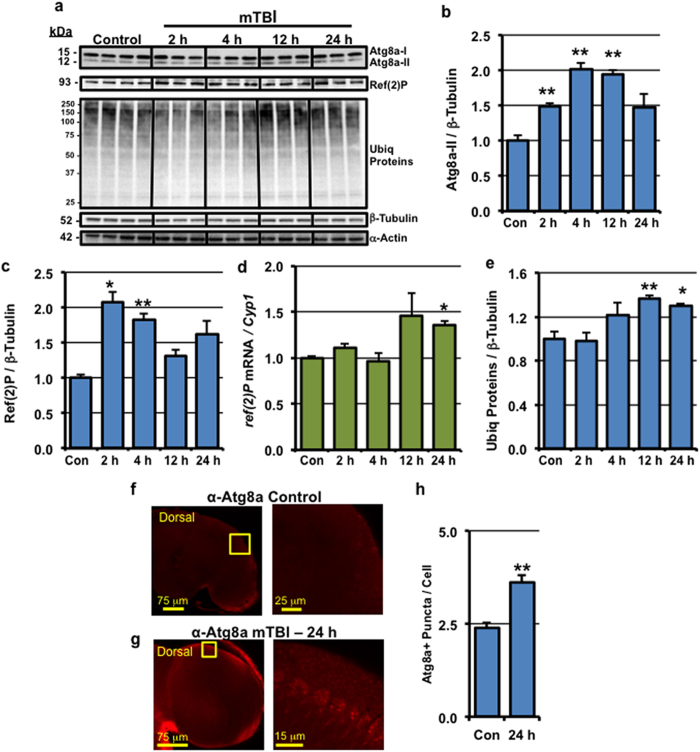
Acute impact of mild TBI on autophagic responses. Replicate cohorts of wild-type male flies were exposed to **10×** mTBI (2.1 m/s intensity) bouts at 1-week of age and collected at indicated time points. (**a**) Western blots of neural extracts were probed sequentially for the Atg8a, Ref(2)P, Ubiquitin (Ubiq) proteins, α-Actin, and β-Tubulin proteins. (**b,c,e**) Quantification of the results from (**a**) normalized to β-Tubulin levels. (**d**) *ref*(2)*P* mRNA expression of control and mTBI-treated flies at the indicated time points (cohorts of 25 flies; n = 3). (**f,g**) The brains from control (n = 15) and **10×** mTBI-treated (2.1 m/s intensity) adult wild-type male flies (n = 10) were dissected at 24 hours post-injury. Representative and enlarged images (highlighted inset) of Atg8a staining patterns are shown in the optic lobe region of the adult CNS. (**h**) Atg8a-positive (Atg8a+) puncta were counted in neuronal cells found in the optic lobe region of the brain from images collected from (**f**,**g**). *P < 0.05, **P < 0.01.

Along with alterations to neurons and the activation of inflammatory systems, there has been growing interest in the roles that intracellular clearance/degradation pathways play following TBI. Recent mammalian TBI studies have highlighted a role of the autophagy pathway following injury^11^,^14^,^35^,^36^. Autophagy involves the *de novo* formation of cytoplasmic vesicles termed autophagosomes, which can engulf and sequester damaged or superfluous cellular material. Autophagosomes are then trafficked to and fuse with lysosomes where the cargo is broken down and recycled back for use by the cell^12^,^37^. The Atg8/MAP-LC3 family of proteins is essential for this process and is widely used as a marker of the autophagic pathway. When autophagy is activated, cytoplasmic LC3-I is lipidated to produce LC3-II, which is the autophagosome-associated version of the protein. This conversion can be detected via Western blots or by formation of LC3-II positive autophagic vesicles, or puncta, in imaging studies^38^. In *Drosophila*, the Atg8a protein (MAP-LC3 homolog) is converted from Atg8a-I (15 kDa) to Atg8a-II (12 kDa) when the pathway is activated and accumulates in to autophagosomes^39^,^40^. Following mTBI (10×, 2.1 m/s), we observed an immediate increase in Atg8a-II levels in the neural tissue that peaked at 4 hours post-injury (Fig. 5a,b). Consistent with these data was the significant increase in the number of Atg8a-positive puncta detected throughout the fly nervous system 24 hours after mTBI (Fig. 5f,g), which correlated with a 50% increase in Atg8a positive puncta, or autophagic vesicles (Fig. 5h).

The build-up of Atg8a-II and autophagosomes following traumatic injury can represent enhanced autophagy levels or a block in the lysosomal degradation of autophagosomes^38^. The Ref(2)P protein, the fly homologue of mammalian p62/SQSTM1, serves as a molecular adapter that targets ubiquitinated proteins for sequestration by autophagosomes^41^. Our work and that of others have demonstrated an *in vivo* role for Ref(2)P in the clearance of ubiquitinated proteins in the fly CNS^41^,^42^,^43^. When autophagy is activated, the Ref(2)P protein is included in autophagosomes and degraded by the lysosome^44^. Therefore, the build-up of Ref(2)P is often used as a marker for impaired autophagic degradation or flux of material through the system^44^. Following traumatic injury, we observed an immediate build-up of Ref(2)P, which gradually decreased over 24 hours (Fig. 5a,c). This increase was independent of an increase in *ref*(2)*P* mRNA expression during that time (Fig. 5d). Ubiquitinated protein levels also gradually increased and peaked at 12 hours post-injury (Fig. 5a,e). Collectively, these results indicate that mTBI causes transient defects in the clearance of autophagosomes in the *Drosophila* brain. These data are consistent with previous studies in mice that demonstrated a block in autophagic flux leading to a build-up of ubiquitinated proteins and p62/SQSTM1, within one day of controlled cortical impact TBI exposure^14^.

**Figure 6:**
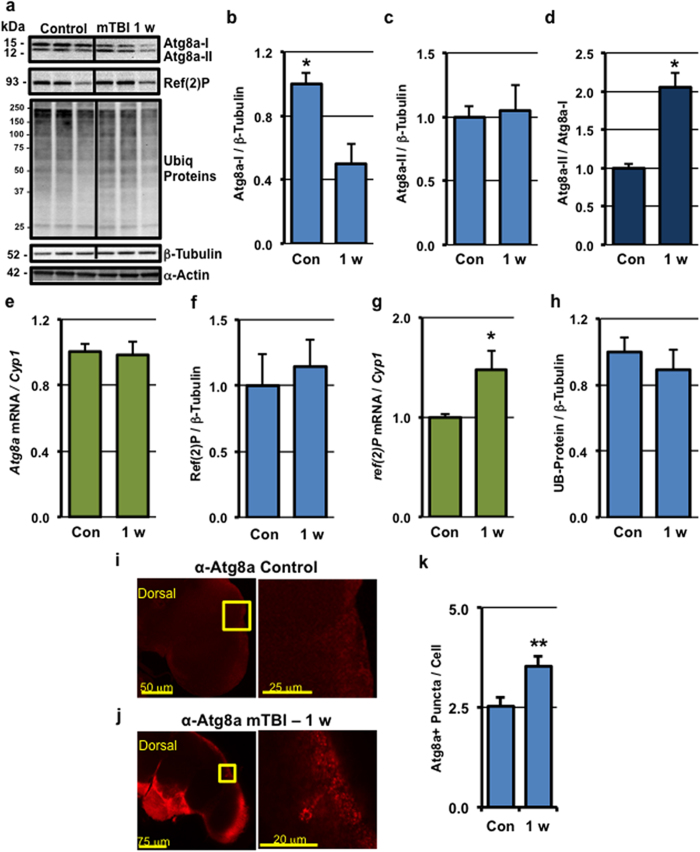
Long-term impact of mild TBI on autophagic responses. (**a**) Replicate cohorts of wild-type flies were exposed to **10×** mTBI (2.1 m/s intensity) bouts at 1-week of age and collected 1-week post-injury (1 w). Western blots of neural extracts were probed for Atg8a, Ref(2)P, Ubiquitin (Ubiq) protiens, α-Actin, and β-Tubulin. (**b,c,f,h**) Quantification of the results from (**a**) normalized to β-Tubulin. (**d**) The ratio of Atg8a-II protein levels to Atg8a-I. (**e,g**) *Atg8a* and *ref*(2)*P* mRNA expression of control and mTBI-treated flies at the indicated time points, respectively (cohorts of 25 flies; n = 3). (**I,j**) The brains of adult male wild-type flies exposed to **10×** mTBI (2.1 m/s intensity) bouts 1-week following injury (n = 12) and age-matched control flies (2-weeks of age, n = 15) were dissected. Representative and enlarged images (highlighted inset) of Atg8a staining patterns are shown. (**k**) Atg8a-positive (Atg8a+) puncta were counted in neuronal cells found in the optic lobe region of the brain from images collected from (**I,j**). *P < 0.05, **P < 0.01.

To determine the long-term impact of traumatic injury on autophagy, young flies were subjected to mTBI injury (10×, 2.1 m/s) and autophagic profiles examined 1-week following injury. Initially the conversion of Atg8a-I to Atg8a-II was assessed in mTBI treated tissues. There was a significant decrease in Atg8a-I levels, although Atg8a-II levels were largely unchanged (Fig. 6a–c), which resulted in an increase in the Atg8a-II/Atg8a-I ratio in mTBI flies, 1-week post-injury (Fig. 6d). The overall decrease in Atg8a protein levels and the lack of change in *Atg8a* expression profiles from control and mTBI treated flies (Fig. 6e) suggested that the conversion of Atg8a-I to Atg8a-II and clearance of autophagosomes by the lysosome was highly active. Consistent with these data, there was a 40% increase in Atg8a-positive puncta in the fly CNS 1-week following the injury when compared to age-matched controls (Fig. 6i–k). In contrast, ubiquitinated proteins and Ref(2)P levels did not differ between control and traumatically injured flies (Fig. 6a,f,h), although there was an increase in *ref*(2)*P* gene expression (Fig. 6g). Combined, these data suggest that the degradation or turnover rate of Ref(2)P may have remained elevated and that autophagy was systemically activated in the nervous system in mTBI-treated flies, 1-week following traumatic injury.

### Sleep alterations in *Drosophila* following mTBI

**Figure 7:**
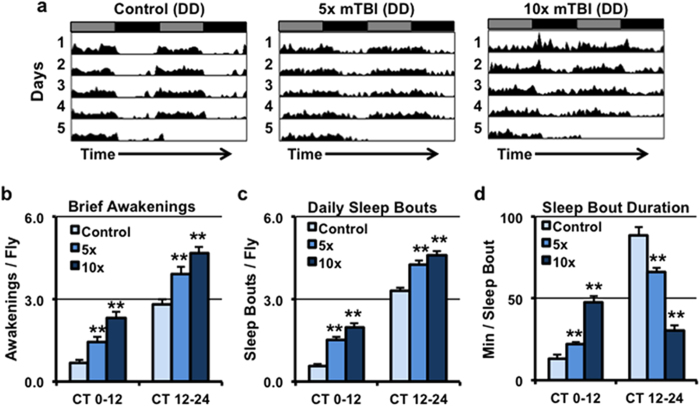
Long-term impact of mild TBI on *Drosophila* circadian and sleep behaviors. Groups of wild-type female flies were exposed to **5×** or **10×** mTBI (2.1 m/s intensity) bouts at 1-week of age and allowed to recover for 5-days before being placed into the DAM system and assessed in constant darkness (DD). (**a**) Representative double-plotted actograms of control (n = 58), 5× (n = 56), and 10× (n = 59) mTBI treated flies. Using the MATLAB-based software, analysis of sleep-related behaviors was performed to assess the number of (**b**) Brief Awakenings, (**c**) Daily Sleep Bouts, and (**d**) Sleep Bout Duration during the subjective day (CT0-12) and subjective night (CT12-24) time periods. **P < 0.01.

Multiple studies have demonstrated that behavioral defects can arise in both humans and mice following repetitive mild TBI^4^,^7^,^45^,^46^. For example, this type of trauma results in sleep maintenance insomnia and alterations in circadian locomotor activity^47^,^48^,^49^. Thus, we investigated whether mTBI causes similar changes in the sleep/wake cycle in *Drosophila*, which is a well-established model organism for studying the molecular and anatomical bases of circadian clock function and the regulation of sleep^50^,^51^. Representative actograms (30-min bins) for flies from each of three different treatment conditions are illustrated in Fig. 7a. Non-injured control flies generally maintained a rhythmic pattern of activity, moving throughout the subjective day and sleeping throughout the subjective night (Fig. 7a). In contrast, many of the mTBI-treated flies did not exhibit a rhythmic pattern of activity (Fig. 7a); there was a dose-dependent increase in the number of arrhythmic flies subjected to 5× (41%) and 10× (56%) mTBI bouts (Supplementary Table S2). Importantly, decreased rhythmicity under the 5× mTBI condition was not accompanied by changes in overall locomotor activity (Supplementary Fig. S4). Furthermore, neither mTBI condition significantly altered total daily sleep or arousal threshold for waking (Supplementary Fig. S4). However, further analysis of sleep behavior profiles highlighted dose-dependent changes to activity and sleep patterns, suggesting poor consolidation of both behaviors. mTBI-treated flies exhibited a significant increase in the number of brief awakenings and sleep bouts during both subjective day (CT0-12) and subjective night (CT12-24) time periods (Fig. 7b,c). These changes were accompanied by a significant reduction in the duration of individual sleep bouts during the subjective night (CT12-24, Fig. 7d). Collectively, these data demonstrate that mTBI exposure results in highly fragmented sleep during the “nighttime” period when sleep would normally be expected to be consolidated. Notably, the sleep maintenance defects observed in mTBI-treated flies are also common features seen in people that have been exposed to head trauma^48^,^49^.

## Discussion

Individual responses and eventual outcomes following TBI are complex and often highly heterogeneous. This is due, at least in part, to the plethora of causes, and varying impact intensities, of TBI. *Drosophila* are extensively used as an *in vivo* model to identify the function of genes involved in multiple human neurodegenerative diseases. Therefore, we hypothesized that *Drosophila* would be a useful model to study the molecular mechanisms and identify novel genetic and environmental factors that influence TBI outcomes. Indeed, recent studies have demonstrated that flies can also be used to effectively model high-impact traumatic injury to neural and intestinal tissues^17^,^52^. In this report, we characterize the development of a complementary injury technique that permits the delivery of highly reproducible levels of both mild-repetitive TBI and severe trauma to the adult *Drosophila* brain. Using the Bead Ruptor-24 tissue homogenizer system, we identified key features typically seen in mammalian TBI models, including increased injury sensitivity in older *Drosophila* cohorts (Supplementary Fig. S1), the activation of the innate immune system (Fig. 4a), elevated Tau phosphorylation profiles (Fig. 2e,f), and alterations to key markers of autophagy (Figs 5 and 6). In addition, to our knowledge, these are the first studies to demonstrate that trauma in *Drosophila* can alter sleep/cycle-related behaviors (Fig. 7), which recapitulate impairments in sleep patterns observed with TBI in mammalian systems.

Previous studies have highlighted the feasibility of using *Drosophila* to model traumatic injuries^17^,^18^. Unlike our Bead Ruptor model, Katzenberger and colleagues developed a cost-effective spring-based “high-impact trauma” (HIT) device^17^. The HIT device requires the experimenter to deflect a spring at a 90° angle to impart trauma to groups of flies^17^. In contrast, our model eliminates several potential points of human error by using a fully programmable and automated system to inflict reproducible levels of injury. Although each injury system uses unique methods of delivering trauma, there were similar findings between these models. Flies subjected to either of these injury regimens resulted in decreased longevity (Fig. 1e) and an increased sensitivity to injury with age (Supplementary Fig. S1), although the flies appeared externally normal (Fig. 1c) and maintained their climbing abilities following trauma (Fig. 1d)^17^. One of the key differences between our model and the Katzenberger model is that there was 20–25% mortality rate in adult flies using the HIT device^17^,^18^. In contrast, mTBI-treated flies (10×, 2.1 m/s) using our model exhibited minimal mortality (Fig. 1b), which is consistent with the minimal mortality of individuals who have sustained a concussion or mild TBI event. Furthermore, our findings demonstrated that mTBI-treated flies had an acute, robust induction of AMP expression profiles (Fig. 4a). The expression peaked within four hours following injury, which is in agreement with the data obtained using the HIT device method^17^. However, the amplitude of AMP gene expression was significantly higher using the mild repetitive injury system outlined in this study^17^,^18^. This is likely reflects a nearly 100% survival rate for flies subjected to our mTBI conditions (Fig. 1b) and thus were able to effectively respond to injury by increasing the expression of AMP genes.

In addition, there was a key difference in trauma-induced intestinal barrier dysfunction between the two TBI methods. Using the HIT device, nearly 25% of *w*^*1118*^ flies had intestinal barrier dysfunction within 24 hours, which directly correlated with the nearly 25% mortality (correlation coefficient of 1.0)^18^. In contrast, <1% of mTBI (10×, 2.1 m/s) treated flies exhibited the “Smurf” phenotype, which was reflective in the minimal mortality during this time (Figs 1b and 3b). Interestingly, under our sTBI regimen, the percentage of flies that had intestinal barrier dysfunction within 24 hours was similar to the mortality rate during that time (Figs 1a and 3b), consistent with the previous Drosophila TBI studies^18^.

The sleep/wake cycle is controlled by highly regulated and conserved processes that are modulated by intrinsic neuronal and extrinsic environmental cues (i.e. light, feeding)^50^. These processes appear to be affected by trauma since mTBI has been reported to alter circadian rhythm and to promote sleep maintenance insomnia and excessive daytime sleepiness^47^,^48^. Interestingly, our *Drosophila* mTBI model was able to recapitulate these observations. One week following injury, mTBI-treated flies showed a marked increase in the number of brief awakenings, especially during the nighttime period, while the duration of each nighttime sleep bout was significantly reduced (Fig. 7b,d). This was coincident with an increase in number of sleep bouts and the duration of the sleep bouts during the subjective daytime period (Fig. 7c,d). Furthermore, there was an increase in locomotor arrhythmia flies following mTBI (Fig. 7a, Supplementary Table S2). A potential contributing factor may be the aberrant production or release of the neuropeptide PDF, which is normally released by clock neurons to promote arousal and signal time of day to other brain regions^24^. Consistent with this hypothesis, we observed a disruption of PDF expressing axonal processes in central clock neurons (large ventral lateral neurons) following TBI (Fig. 2a–d), suggesting that the release of PDF is likely compromised.

Sleep impairments can be detrimental to the long-term health of organisms^53^. Indeed, several studies have demonstrated that sleep deprivation results displayed in pro-inflammatory cytokine production^53^. Coincidently, 1-week following trauma, flies displayed impairments in sleep consolidation have increased expression of innate immune genes (AMPs, Fig. 4c). These results suggest that sleep defects could be a contributing factor to the systemic inflammation observed in mTBI-treated flies 1-week following injury. Whether this is the case or whether long-term sleep defects contribute to the increased brain inflammation found in TBI patients remains to be explored.

In addition to impacting the behaviors and the innate immune system, our mTBI regimen altered the profiles of key autophagy markers. Following exposure to mTBI (10×, 2.1 m/s intensity), there was a build-up of Atg8a-II, Ref(2)P, and ubiquitinated proteins in neural tissues within 24 hours following injury (Fig. 5a–c,e). Further, we detected elevated Atg8a positive vesicles, or puncta, indicating autophagosome formation occurs throughout *Drosophila* brain (Fig. 5f–h)^39^,^40^. Collectively, these results suggest that the damage caused by mTBI results in a transient block in the clearance of autophagosomes in neural tissues. These data are consistent with mouse studies using the controlled cortical impact TBI model, which demonstrated an impairment of lysosomal function and a block in autophagic flux^14^.

In summary, the *Drosophila* mTBI model detailed in this report recapitulates multiple aspects of mammalian physiology following injury, indicating that flies can be used to model and identify the genetic, environmental and age-dependent factors that influence TBI outcomes. Ultimately, using the *Drosophila* mTBI model would allow for the rapid identification of key genes, pathways, and environmental factors that facilitate cellular and tissue repair and could aid in the identification and design of potential therapeutics, or treatments, that could promote the survival and healing of neuronal tissues.

## Methods

### Drosophila Stocks and Culturing Conditions

The Canton-S, *w*^*1118*^ and APPL-Gal4 stock lines have been described previously^21^. The PUAS-Tau.wt1.13 (BL#-51362, G. Jackson) line was obtained from the Bloomington Stock center (Bloomington, flybase.org)^26^. Crosses between Canton-S (CS) females and *w*^*1118*^ males (*w*^*1118*^/+) generated F1 offspring were used as wild-type (WT) controls for all experiments. Male and female flies were collected and maintained in same-sex cohorts (25 flies per vial) on standard lab media (agar, molasses, yeast, cornmeal, propionic acid, nipagin) and culturing conditions at 25 °C in 50–60% humidity^21^. For circadian and sleep studies all flies were initially entrained using a standard 12 h:12 h light:dark cycle (**LD**).

### Traumatic Brain Injury

Flies were incapacitated using CO_2_ and placed in a clean 2-ml screw cap tube (10 flies/tube). To eliminate any confounding effects of CO_2_ exposure and the stress of being placed in a 2-ml tube, both control (non-injured) and TBI treated fly cohorts were subjected to the above conditions. For injury, tubes containing flies were placed into the Omni Bead Ruptor-24 homogenizer (Omni International, Kennesaw, GA, USA) and subjected to specific pre-programmed shaking conditions. This instrument permits highly controlled shaking conditions that include a broad range of intensities (0.8–8.0 m/s range). Flies were subjected to a particular intensity (m/s) injury for 5 seconds. For multi-bout conditions, fly cohorts were injured (5 seconds) and allowed to recover for 30 seconds before the start of subsequent injury bouts. Following the completion of the injury, all fly cohorts were put into vials, which were placed on their sides to allow for a full recovery.

### Video imaging and measurements of impact energy

High-speed videos were taken using a Nikon 1 camera to assess the type of injury sustained by flies, at Omni International. During a single 5-second injury bout, flies collided with the tube wall multiple times. With each Bead Ruptor rotation, the flies experienced approximately 2 impacts (Supplementary Video). On closer examination, we observed that each fly body segment (head, thorax, abdomen) made contact with the tube multiple times. To simplify calculations, a single fly was placed in a 2-ml tube marked with distance delineations so that the velocity of the fly could be calculated by correlating the distance traveled to the frame rate of the camera. Based upon the estimated mass of adult male flies (~0.8 mg), the amount of kinetic energy exerted on each animal could be calculated for a single mTBI bout (2.1 m/s intensity for 5 seconds). The amount of kinetic energy imparted onto the fly was calculated using equation (1), where EK = kinetic energy [joules], m = mass [g], v = velocity [m/s]). Male flies were shown to travel on average between 1.4–2.1 m/s at the time of impact to the tube’s wall. During a 5-second bout, each fly sustained approximately 215 impacts. Thus, for every 5-second injury bout at 2.1 m/s intensity, flies would be expected to absorb between 175 and 395 μjoules of energy.
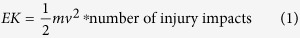


### Mortality and Longevity Profiles

Mortality was determined by subjecting 1-week old male flies to a single 5-second injury at different intensities and determining the percent of dead flies after 24 hours. During this 24 hour time-period, all of the non-injured control flies survived. The longevity profiles of flies were determined after subjecting the flies to sTBI (1× at 4.35 m/s) or mTBI (5×, 10× at 2.1 m/s) conditions at 1 or 3-weeks of age and the number of dead flies were recorded 3 times each week.

### Negative Geotaxis Response

The *Drosophila* negative geotaxis response involves the mechanical stimulation of an innate escape response^20^. The rapid iterative negative geotaxis (**RING**) protocol and apparatus has been described previously^21^. Groups of 25 male flies (1 week of age) were tapped down and digital images taken after 5 seconds. Flies were allowed to rest for 1-min between a total of two replicate runs. The digital images were analyzed and each fly was scored as to the distance traveled, with values ranging from 0 (bottom) to 6 (top). Replicate runs were used to establish the average climbing index or ability for each fly cohort.

### Intestinal Barrier Dysfunction

Following indicated TBI conditions, male flies were placed on a standard lab media and BLUE dye No. 1 (2.5% wt/vol, ThermoFisher Scientific, Grand Island, NY, USA). After 24 hours, the percentage of flies that had blue dye dispersed throughout their body was counted and used to determine the percentage of “Smurf” flies (see Fig. 3a).

### Quantitative PCR

Male flies (1-week) were subjected to 10× mTBI (2.1 m/s) bouts, collected at defined time-points, flash frozen and stored (−80 °C). A minimum of three independent biological samples for each condition was used and similar results were obtained from three replicate studies. RNA was isolated from 25 heads using Trizol (ThermoFisher Scientific) and cDNA libraries generated using the RevertAid First Strand cDNA Synthesis kit, with a combination of random hexamer and oligo-dT primers (Thermo Scientific, Pittsburg, PA, USA). Quantitative PCR was performed on a CFX Connect Real-Time PCR Detection System (Bio-Rad, Hercules, CA, USA) and Universal PCR SYBR Mix reagents (Bio-Rad). Primer gene sequences are available upon request. Melt curve analyses of all qPCR products confirmed the production of a single DNA duplex. The Pfaffl method was used to quantitate expression profiles and *Cyp1* used as a reference gene. Relative mRNA levels of non-injured flies were set at 1.0 and subsequent expression levels from different time-points were expressed as normalized values.

### Western Analysis

Male flies (1-week) were subjected to 10× mTBI (2.1 m/s) bouts, collected at defined time-points, flash frozen and stored (−80 °C). A minimum of three independent biological samples for each condition was used and similar results were obtained in replicate studies. Separated heads were extracted with lysis buffer (2% SDS, 150 mM NaCl, 50 mM Tris, pH 7.5) containing protease inhibitors (Thermo Scientific/Pierce, Rockford, IL, USA) using the Bead Ruptor-24 System (Omni International). Cellular debris was removed (10,000 × *g*, 10 min) and the protein concentrations of supernatants were determined using the DC Protein assay (Bio-Rad). Protein samples (20 μg) were resolved on a 12% Bis-Tris gel (Bio-Rad) and transferred onto Immobilon-P PVDF membranes (Millipore Corp., Billerica, MA, USA) using the Trans-Blot Turbo system (Bio-Rad). Blots were probed using various antibodies at 1:500–2,000 dilutions overnight, at 4 °C. Blots were developed using Thermo Scientific West Dura Substrate (Thermo Scientific/Pierce) and the ChemiDoc digital Imaging System and Quantity One software (Bio-Rad). Protein bands were quantified using ImageJ software (imagej.nih.gov/ij/). Antibodies used for these studies include anti-α-Actin (JLA20, Developmental Studies Hybridoma Bank [DSHB], Iowa City, Iowa, USA), anti-Bruchpilot (nc82, DSHB), anti-Discs large (4F3, DSHB), anti-GABARAP (Atg8a, E1J4E, Cell Signaling Technology [CST], Danvers, MA, USA), anti-Ref(2)P^21^, anti-SYNORF1 (Synapsin, 3c11, DSHB), anti-Syntaxin (8C3, DSHB), anti-phospho-Tau (AT8, ThermoFisher Scientific), anti-Tau (T46, ThermoFisher Scientific), anti-β-Tubulin (E7, DSHB), and anti-Ubiquitin (P4D1, CST). The specificities of the anti-phospho-Tau and anti-Tau antibodies are shown in Supplementary Fig. S5. The calculated molecular weight of 63 kDa in these studies is similar to previously published results where various isoforms of human Tau was expressed in Drosophila neurons (60–62 kDa)^26^. The specificity of the anti-Ref(2)P antibody is shown in Supplementary Fig. S6 21. The anti-GABARAP monoclonal antibody recognizes two protein bands at 15 kDa and 12 kDa (Supplementary Fig. S7) that correspond to the *Drosophila* Atg8a-I and Atg8a-II proteins respectively^39^. All Western blot images and quantitative comparisons of respective proteins were from samples that were run on the same gel.

### *Imaging* Studies

Male flies at 1-week of age were exposed to mTBI (10×, 2.1 m/s) conditions and were collected at specific time-points following injury. Brains were dissected from surrounding cuticle and fixed for 45 min on ice in 4% PFA-PBS, washed 3 times (1× PBS, 0.1% Triton X-100) and blocked in 5% normal goat serum (NGS, Jackson ImmunoReseach Laboratories, Inc., West Grove, PA, USA). Samples were incubated overnight at 4 °C with anti-PDF (1:500 dilution, c7, DSHB), anti-ELAV (1:500 dilution, 9F8A9, DSHB) and/or anti-GABARAP antibodies (1:250 dilution, E1J4E, CST) as indicated. Washed samples were blocked (5% NGS) and incubated for 3 hours at room temperature with Alexa Fluor-488 (1:250 anti-mouse) or Cy3 (1:250 anti-rabbit) secondary antibodies (Jackson ImmunoReseach Labs, Inc.) as previously described^29^,^54^. Brains were imaged using either a Leica SP8 or a Zeiss 710 confocal microscope. To assess levels of neuronal damage, brains dissected from control (n = 15) or mTBI (10×, 2.1 m/s) exposed flies at 1 day (n = 11) or 5 days (n = 9) following injury were stained using anti-PDF and Alexa Fluor-488 secondary antibodies. The PDF peptide hormone staining patterns of individual adult optic lobes were obtained from multiple confocal Z series of images. To quantify PDF fluorescence, the perimeters of adult optic lobes (medullary ganglia) were outlined, excluding the soma of PDF expressing neurons, and measured for their PDF fluorescence intensity using ImageJ software. The PDF signal was subjected to a threshold to prevent the inclusion of background signal in the analysis (Supplementary Fig. S2). For autophagic puncta assessment, male flies were collected at one day (n = 10) or seven days (n = 12) following injury, along with age-matched non-injured control flies (n = 15). Brains were stained using anti-Atg8a (Cy3) and anti-ELAV (Alexa Fluor-488) antibodies. The average number of Atg8a positive puncta per ELAV-positive cortical neuron was determined from counting the number of Atg8a puncta in multiple 10 μm^2^ image fields from different adult Drosophila CNS cohorts. On average, a 10 μm^2^ area of the adult Drosophila cortex contained approximately 4.4 neuronal cell bodies. A minimum of ten 10 μm^2^ area fields for each individual brain were used for the calculations of Atg8a-positive puncta per cell. Thus, over 100 cells were counted per condition. For additional information regarding autophagosome puncta counts, see Supplementary Fig. S8.

### Sleep and Arousal Analysis

Female flies (1-week) were subjected to 5× or 10× mTBI bouts (2.1 m/s) and allowed to recover under standard 12 h:12 h LD cycle conditions for 5 days. For sleep analysis, individual flies were transferred to tubes (5 mm × 65 mm) containing fly media and placed into a DAM5 System (Trikinetics Inc., Waltham, MA, USA). Flies were allowed to recover and acclimate for an additional 2-days under 12 h:12 h LD cycle conditions before being assayed in constant dark (**DD**) conditions. Movement or locomotor activity was recorded as the number of infrared beam breaks that were collected in 1-minute bins for 6-days using DAM system v3.8 software. Data sets were further analyzed using the Sleep-Lab software developed by Dr. William Joiner, as previously described^54^. Flies that died during the sleep study periods were removed from analysis. The waking activity, brief awakenings, daily sleep bouts, and sleep bout duration were calculated using the Sleep-Lab software. Brief awakenings refers to 5-min periods with four or fewer activity counts that were preceded and succeeded by a minimum of 5 minutes of inactivity^55^. Arousal measurements were carried out at 25 °C using 12 h:12 h LD cycle conditions using DAM5 monitors (Trikinetics) placed in a custom-built programmable shaking apparatus, which permits variable one-dimensional oscillations at increasing frequencies. Direction of movement is perpendicular to the direction of activity tubes to avoid movement artifacts and occur at 30-min intervals between ZT17-19 (dark time-period). Previously, 30-min intervals between oscillations were found to be sufficient for all responsive flies to fall asleep. However, any flies moving across the infrared beam 10 minutes prior to stimulation were excluded from the analyses. Percent responsiveness was calculated as the fraction of the remaining flies moving within 5-min of stimulation and was averaged over 3 successive days for each experimental run.

### Statistical Analysis

Statistical analyses between control and injured groups were performed using the Student’s T-test (two-tailed, unpaired). All values are reported as means + SEM.

## Additional Information

**How to cite this article**: Barekat, A. *et al*. Using *Drosophila* as an integrated model to study mild repetitive traumatic brain injury. *Sci. Rep.* **6**, 25252; doi: 10.1038/srep25252 (2016).

## Supplementary Material

Supplementary Information

Supplementary Video
