# Supplementary material for: Using Drosophila as an integrated model to study mild repetitive traumatic brain injury
Source: Sci Rep. 2016 May 4;6:25252. doi: 10.1038/srep25252 (PMC4855207; doi:10.1038/srep25252)
Supplement: Supplementary Information [file srep25252-s1.pdf]

Using *Drosophila* as an integrated model to study mild repetitive traumatic brain injury

Ayeh Barekat[1,2], Arysa Gonzalez[1], Ruth E. Mauntz[1], Roxanne W. Kotzebue[1,2], Brandon Molina[1,2], Nadja El-Mecharrafie[1,2], Catherine J. Conner[2], Shannon Garza[2], Girish C. Melkani[2], William J. Joiner[3], Marta M. Lipinski[4], Kim D. Finley[1,2,5,*] and Eric P. Ratliff[1,2,5,*]

[1]Donald P. Shiley BioScience Center, San Diego State University, San Diego, CA, USA. [2]Department of Biology, San Diego State University, San Diego, CA, USA. [3]Department of Pharmacology, University of California San Diego, La Jolla, CA, USA. [4]Shock, Trauma, and Anesthesiology Research (STAR) Center; Department of Anesthesiology, University of Maryland School of Medicine, Baltimore, MD, USA. [5]Department of Chemistry, San Diego State University, San Diego, CA, USA.
[*]Correspondence should be addressed to K.D.F. (kfinley@mail.sdsu.edu) or E.P.R. (eratliff@mail.sdsu.edu).

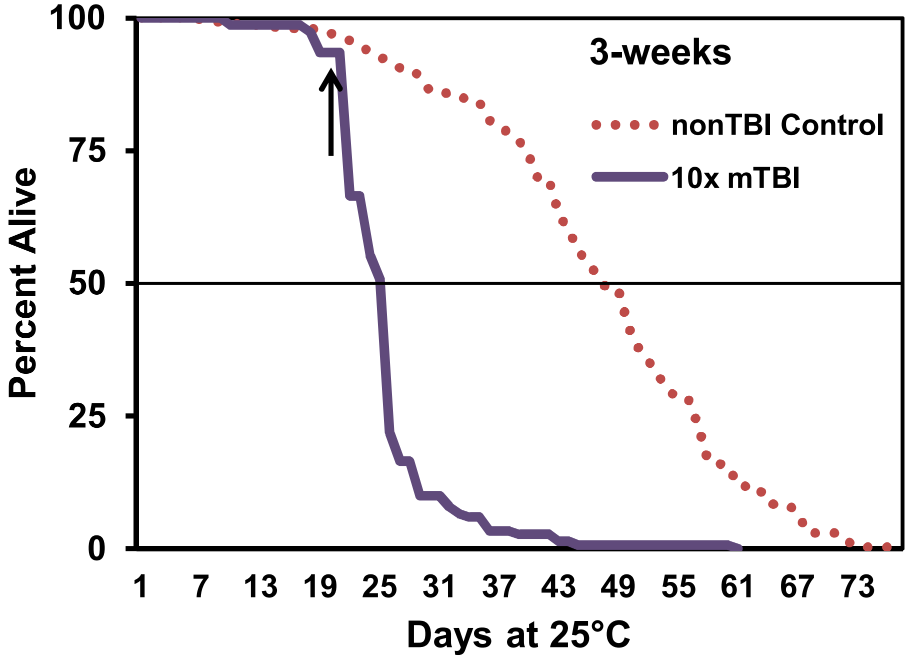

Supplementary Figure S1. Lifespan profiles of flies treated with mild TBI at 3-weeks of age. Lifespan profiles of wild type male controls (n=382) and flies (n=152) exposed to 10x mTBI bouts (2.1 m/s) at 3-weeks of age (arrow indicates time of injury). Unlike young adults (1-week), older male flies exposed to 10x mTBI injury bouts showed an immediate decline in viability and did not exhibit the 2-week delay in mortality when exposed to mild levels of trauma. See Supplementary **Table S1** for additional details.

α-PDF Control α-PDF mTBI – 5 d

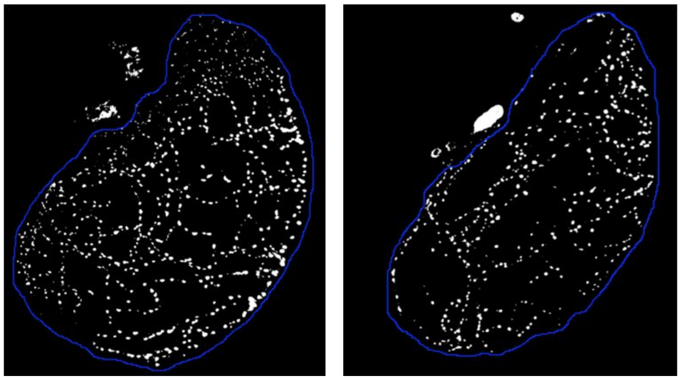

Supplementary Figure S2. **Quantification of disruption to PDF positive neurons and structures due to trauma**. Representative compressed images of outlined individual optic lobes (blue line) from control (n = 15) and mTBI-treated (10x, 2.1 m/s; n = 9) adult male fly brains. PDF intensity was measured within each defined area that excluded the soma of l-LN_V neurons. These were used to highlight changes to PDF positive projections and synapses within each optic lobe.

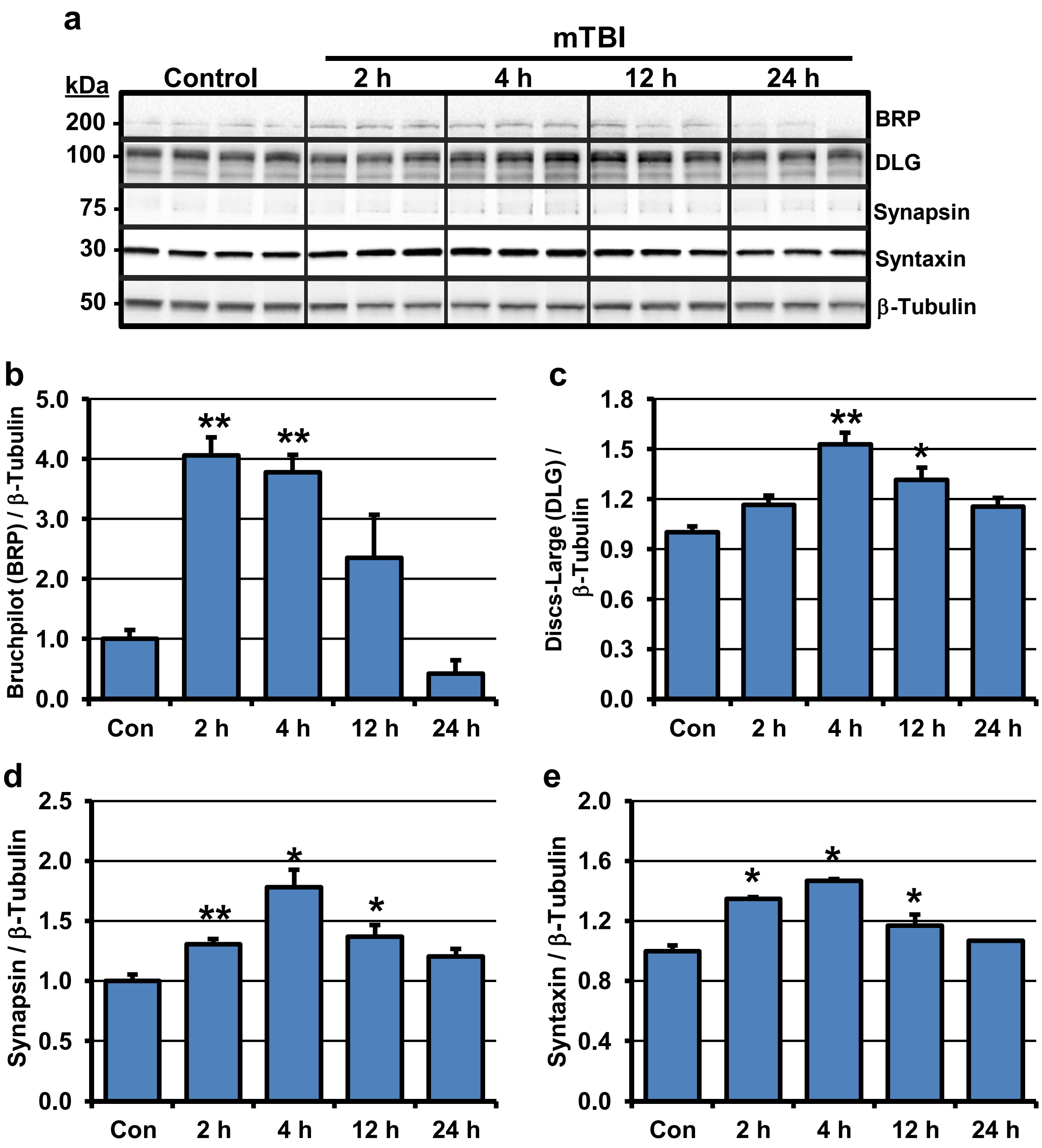

Supplementary Figure S3. Alterations in synaptic protein levels. Triplicate cohorts of wild-type male flies (25 flies per sample) were exposed to 10x mTBI bouts (2.1 m/s). (**a**) Western blots of endogenous *Drosophila* synaptic proteins Bruchpilot (**BRP**), Discs large (**DLG**), Synapsin, Syntaxin, and β-Tubulin from neural lysates of control and mTBI flies. Quantification of (**b**) Bruchpilot (BRP), (**c**) Discs large (DLG), (**d**) Synapsin and (**e**) Syntaxin proteins from the Western blots illustrated in (**a**). All individual protein quantifications were normalized to β-Tubulin. * P ≤ 0.05, ** P ≤ 0.01.

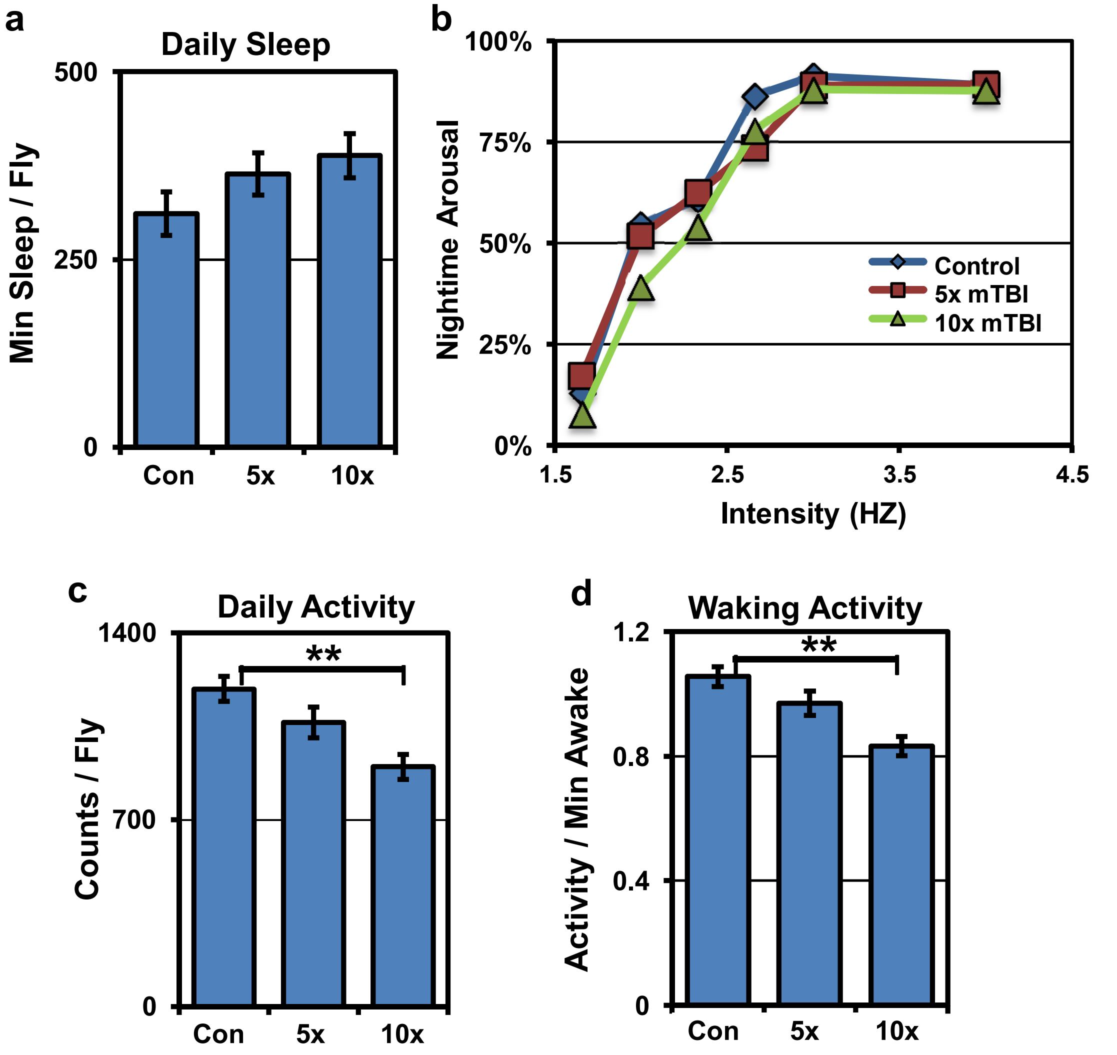

Supplementary Figure S4. Sleep, activity and arousal thresholds of mTBI treated flies. Groups of wild-type female flies were exposed to **5x** or **10x** mTBI bouts (2.1 m/s) and allowed to recover and entrain for 5 days under standard 12 h:12 h light:dark conditions (**LD**). Fly cohorts were assayed using standard DAM systems for 6 days in constant dark conditions (**DD**). (**a**) Daily Sleep (min Sleep / 24 hours / Fly). (**b**) Arousal thresholds were determined for control and injured flies (5-days post mTBI) using DAM systems. The percentage of control, 5x and 10x treated flies aroused after receiving stimuli of varying shaking intensities (HZ) are shown for nighttime periods (ZT12-24). (**c**) The Daily Activity (counts or beam crossing / fly / 24 hours). (**d**) Average Waking Activity (activity / min awake per fly). ** P ≤ 0.01.

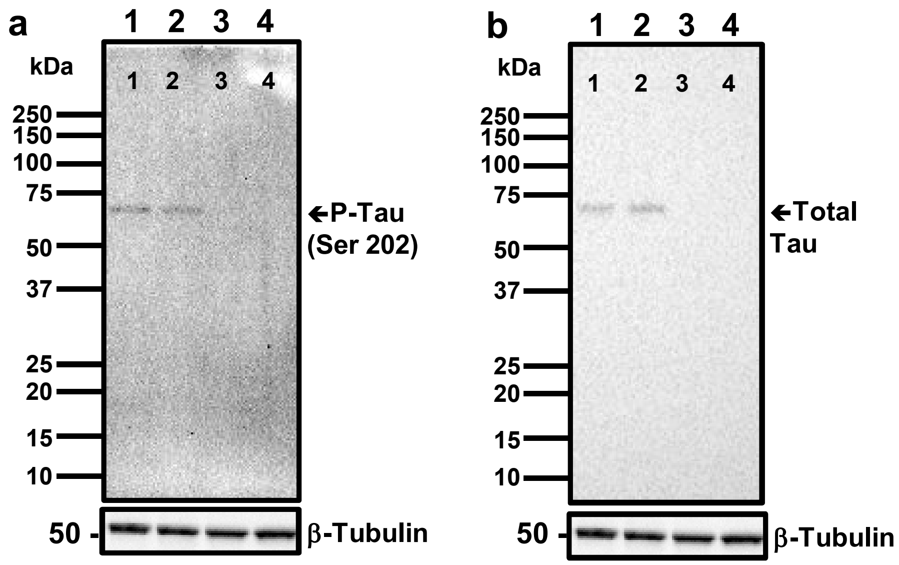

Supplementary Figure S5. Assessment of Tau antibodies. Duplicate neural lysates from female flies expressing hTau in adult neurons (**1-2**, APPL-Gal4/UAS-hTau) and control female fly cohorts (**3-4**, APPL-Gal4/+) were analyzed by Western blot. (**a**) Westerns were probed for the Ser202 phosphorylation of Tau (P-Tau) was measured using the commercially available AT8 antibody (ThermoFisher Scientific). (**b**) Total Tau levels were measured using a second the commercially available T46 antibody (ThermoFisher Scientific). Both of the Tau bands run at approximately 63 kDa and are consistent with other published observations of hTau profiles in *Drosophila* tissues.

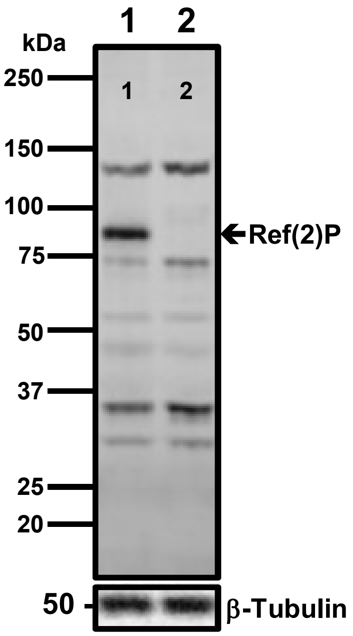

Supplementary Figure S6. Assessment of *Drosophila* Ref(2)P antibody specificity. Neural lysates from **1**) wild-type and **2**) *Ref(2)P*$^{c03993/e00482}$ homozygous mutant male flies were analyzed by Western blot. The 93 kDa protein band that corresponds to the specific full-length Ref(2)P protein is highlighted (arrow).

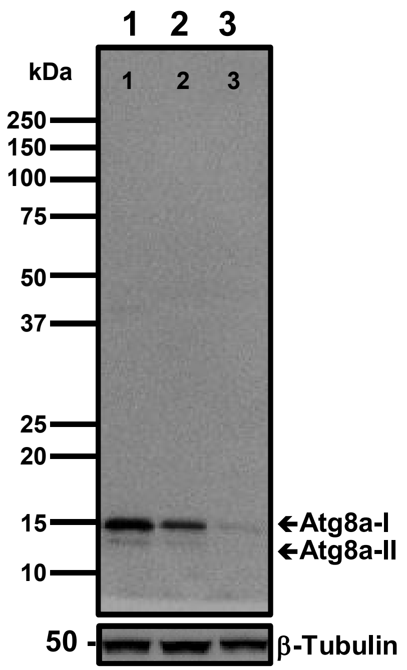

Supplementary Figure S7. Assessment of Atg8a antibody specificity. Neural lysates from **1**) wild-type, **2**) *Atg8a*$^{1/+}$ heterozygous mutant, and **3**) *Atg8a*$^{1/2}$ mutant female flies were analyzed by Western blot. The two protein bands that correspond to the *Drosophila* Atg8a-I (15 kDa) and Atg8a-II (12 kDa) bands are indicated.

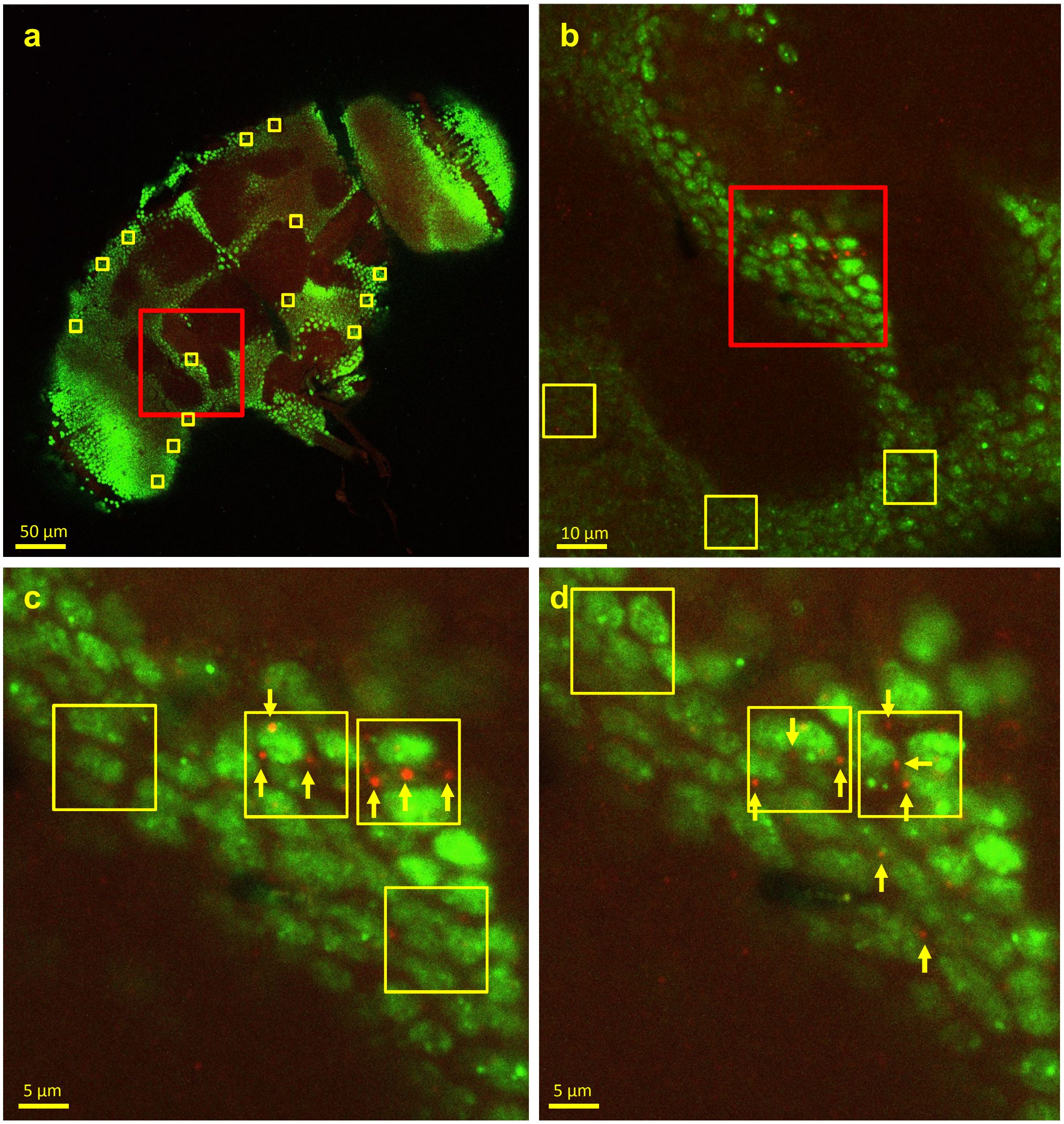

Supplementary Figure S8. Assessment of autophagic puncta in the adult *Drosophila* CNS. Brains from non-injured wild-type (1-week) male flies were dissected, fixed and co-incubated with rabbit anti-Atg8a (autophagosome marker, CST) and mouse anti-ELAV (neural marker, DSHB) antibodies. The soma (cell bodies) of individual neural cells are highlighted in green and the red puncta mark individual autophagosomes (yellow arrows). Yellow boxes represent 10 μm^2 areas in CNS regions where autophagosomes were typically counted. Red boxes highlight areas that are presented in subsequent panels with increased magnification. (**a**) Whole brain image. (**b**) Increased magnification of the area highlighted in (**a**). (**c-d**) Increased magnification of area highlighted in (**b**), taken at differing depths. Given the complex structure of the *Drosophila* CNS, the ELAV staining partly obscured the identification and counting of underlying puncta. Thus, for **Fig. 5f-g** and **Fig. 6i-j**, ELAV staining was not included. A minimum of ten 10 μm^2 area fields for each individual brain were used for the calculations of number of Atg8a-positive puncta per cell.

Supplementary Table S1. Adult Longevity Profiles

	Control	1-week hTBI	1-week mTBI 10x	1-week mTBI 5x	3-week mTBI 10x
**Average (days)**	46.88	19.06**	26.32**	30.87**	25.5**
**SEM**	0.67	0.86	0.45	0.73	0.45
**N**	382	59	285	166	152
**% Change**		-59%	-44%	-34%	-46%

** P < 0.01 compared to control, non-injured flies.

Supplementary Table S2. Circadian Locomotor Activity

	Control	1-week mTBI 5x	1-week mTBI 10x
**Period**	24	24.4	24.8
**SEM**	0.02	0.23	0.43
**n**	58	56	59
**% Arrhythmic**	**21%**	**41%**	**56%**

Supplementary Video. ***Drosophila* mTBI model.** An individual fly was placed in 2-ml screw cap centrifuge tube and subjected to a single mTBI injury bout at 2.1 m/s intensity. High-speed videos were taken at 1200 frames per second using a Nikon 1 camera by staff members at the Omni International Corporation (see Acknowledgements).
